# Observation of a shape resonance of the positronium negative ion

**DOI:** 10.1038/ncomms11060

**Published:** 2016-03-17

**Authors:** Koji Michishio, Tsuneto Kanai, Susumu Kuma, Toshiyuki Azuma, Ken Wada, Izumi Mochizuki, Toshio Hyodo, Akira Yagishita, Yasuyuki Nagashima

**Affiliations:** 1 Department of Physics, Tokyo University of Science, 1-3 Kagurazaka, Shinjuku, Tokyo 162-8601, Japan; 2 Atomic, Molecular and Optical Physics Laboratory, RIKEN, 2-1 Hirosawa, Wako, Saitama 351-0198, Japan; 3 Institute of Materials Structure Science, High Energy Accelerator Research Organization (KEK), 1-1 Oho, Tsukuba, Ibaraki 305-0801, Japan

## Abstract

When an electron binds to its anti-matter counterpart, the positron, it forms the exotic atom positronium (Ps). Ps can further bind to another electron to form the positronium negative ion, Ps^−^ (e^−^e^+^e^−^). Since its constituents are solely point-like particles with the same mass, this system provides an excellent testing ground for the three-body problem in quantum mechanics. While theoretical works on its energy level and dynamics have been performed extensively, experimental investigations of its characteristics have been hampered by the weak ion yield and short annihilation lifetime. Here we report on the laser spectroscopy study of Ps^−^, using a source of efficiently produced ions, generated from the bombardment of slow positrons onto a Na-coated W surface. A strong shape resonance of ^1^P^o^ symmetry has been observed near the Ps (*n*=2) formation threshold. The resonance energy and width measured are in good agreement with the result of three-body calculations.

## Abstract (web summary)

The Positronium negative ion is formed by two electrons bound to a positron, and experimental investigations of its states and energy levels are difficult due to its short lifetime. Here, the authors report on laser spectroscopy of positronium using a source of efficiently produced ions.

## Graphical abstract

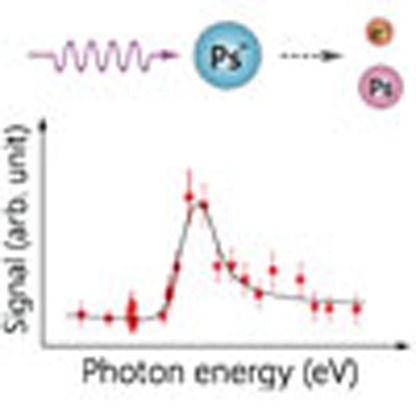

The three-body problem with a Coulomb interaction has been the focus of attention in fundamental physics for not only classical mechanics but also quantum mechanics, since the Schrödinger equation for a three-body system has not been solved analytically, despite the proposal of a variety of approximation approaches. The Ps^−^ ion^1^,^2^ can be regarded, from an atomic and molecular physics perspective, as an intermediate between the two extreme cases of H^−^ (atomic-like) and H_2_^+^ (molecular-like) because of its mass ratio^3^,^4^,^5^. Since the theoretical simplifications applied to atoms or molecules may often be inadequate, research on Ps^−^ structure and dynamics can provide a stringent testing ground for the quantum mechanical three-body problem.

Theoretical studies indicate that Ps^−^ has only a ground state (^1^S^e^) where the two electrons have opposite spins, and no particle-stable excited states^6^,^7^, unlike the H^−^ ion, which has a doubly excited ^3^P^e^ state. However, quasi-bound states (resonances) have been theoretically predicted in the vicinity of the formation thresholds of Ps (for principal quantum number *n*≥2) (ref. 8), offering the expectation that experiments will reveal rich structures around the energy levels of Ps^−^. Although the resonance states spontaneously dissociate into Ps in the ground state or lower-lying excited state and electron in the continuum, interference between the direct detachment process and the detachment via the resonance state gives rise to characteristic structures on the cross sections near the resonance energy. The resonance of the ^1^P^o^ symmetry, which is accessible by the single-photon absorption of Ps^−^, has been theoretically investigated^9^,^10^,^11^,^12^,^13^. In the vicinity of the *n*=2 threshold, a strong shape resonance, in which the electron is temporarily trapped by a centrifugal barrier potential, is thought to lie above the level. Moreover, a series of Feshbach resonances, which originates from an attractive dipole potential formed by the 2S−2P degeneracy of Ps (*n*=2), is also expected to lie just below this threshold.

Historically, the existence of Ps^−^ was predicted by Wheeler^1^ in 1946 and was discovered in the laboratory, using the beam-foil method, by Mills^2^ in 1981. Since then numerous theoretical studies have been devoted to exploring the nature of this exotic ion^14^,^15^,^16^,^17^,^18^,^19^,^20^,^21^,^22^,^23^. However, because of the extremely weak ion yield and short annihilation lifetime (479 ps), experimental investigations on Ps^−^ have been limited to a few measurements of its annihilation rate (ref. 24 and references therein). Recently, an efficient formation method for this ion was found where, on impacting slow positron beams onto tungsten (W) surfaces coated with sub-monolayer alkali-metal atoms, the conversion efficiency increased by double digits due to the coating^25^,^26^,^27^. This discovery has opened up new experimental fields for Ps^−^, such as its photodetachment^28^ and the consequent generation of an energy-tunable Ps beam^29^.

In this letter, we report on a study of its kind made on the laser spectroscopy of Ps^−^ ions, generated by this efficient production scheme. We report the observation of a strong shape resonance of ^1^P^o^ near the Ps (*n*=2) formation threshold. The resonance energy and width measured are in good agreement with the result of three-body calculations.

## Result

### Experimental setup and procedure

**Figure 1:**
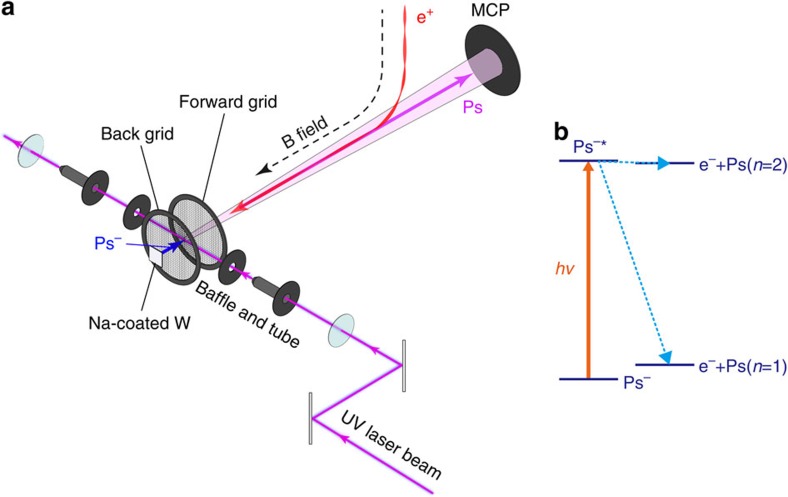
Schematic diagram of the experimental setup and the energy levels of Ps^−^. (**a**) A pulsed slow positron beam is guided along a magnetic field and impacted onto a Na-coated W target to generate Ps^−^ ions. The ions are accelerated by a static electric field between the target and a back grid, and are then irradiated by ultraviolet laser beam in the electric field-free region between the forward and back grids biased at the same voltage. The neutral Ps atoms formed by (resonant) photodetachment are detected by the MCP. (**b**) Optical transition from Ps^−^ (^1^S^e^) to Ps (*n*=1 or 2) +e^−^ continuum state via shape resonance (^1^P^o^) as indicated by Ps^−*^.

A pulsed slow positron beam at the KEK-IMSS slow positron facility^30^ was used to synchronize the Ps^−^ beam and a pulsed ultraviolet laser beam of sufficient photon density for the photodetachment of the short-lived Ps^−^ ions. The positron beam, with a repetition of 50 Hz and pulse-width of 12 ns FWHM, was transported to the measurement chamber with a kinetic energy of 4.2 keV, passing through a plate with a 5 mm circular aperture. The beam intensity and the diameter were 4 × 10^3^ e^+^ per pulse and 4 mm FWHM, respectively. As shown in Fig. 1a, it was deflected by an angle of 45° along a curved magnetic field (∼0.01 T), then passed through forward and back grids biased at the same voltage of 3,400 V and, finally, impacted onto a W target coated with a 0.3 monolayer of Na (Supplementary Note 1). In order to maintain Ps^−^ emission from the surface^26^ for the duration of the runs, the chamber was evacuated to a pressure of 1 × 10^−8^ Pa.

When positrons impinge onto a surface, they can lose their kinetic energies and thermalize in the bulk. Some diffuse back to the surface to form Ps^−^ ions, and these are emitted spontaneously with a low kinetic energy governed by the Ps^−^ affinity (∼−3 eV). The formation efficiency of Ps^−^ ions against the incident positron flux is reported to be about 2% (ref. 26). The Ps^−^ ions formed in this setup were accelerated by the potential difference, *V*, between the target and back grid. The potential of the target was varied to set the value of *V*. The ions intersected the laser beams from a tunable dye laser (see the ‘Methods' section for details on the laser system) at right angle in the electric field-free region between the two grids. The effects of stray magnetic fields in the beam intersection region were considered: for a field of about 3 × 10^−3^ T with a Ps^−^ speed of 0.07*c* (*V*=3,400 V), where *c* is the speed of light, the effective electric field was estimated to be 6 × 10^2^ V cm^−1^. Motional Stark-broadening and shift of resonance energies are small enough to be neglected at this field strength^31^,^32^.

Neutral Ps atoms formed both by the direct photodetachment process and via the resonances (Fig. 1b) were detected by a micro-channel plate (MCP), of effective diameter 42 mm, while charged particles were removed by the curved magnetic field. The residual background was due to stray light, reflected from the laser inlet and outlet fused-silica windows coated by broadband anti-reflection coatings and annihilation γ-rays from the target. In order to reduce the MCP signal due to the stray light, baffles and cylindrical tubes with 5 mm diameter apertures were placed between the target and each window.

*Para*-Ps (*S*=0) and *ortho*-Ps (*S*=1) are formed in the Ps^−^ photodetachment process. As for the S-states, *para*-Ps atoms decay with a lifetime of 125*n*^3^ ps into two γ-rays, while *ortho*-Ps atoms decay with a lifetime of 142*n*^3^ ns into three γ-rays. The 2P-states, which have longer lifetimes against annihilation (0.1–3 ms) (refs 33, 34), are de-excited to 1S-states with a lifetime of 3.2 ns and these then decay according to their own annihilation lifetimes. Owing to the short flight length (<20 mm) of *para*-Ps atoms, even in the *n*=2 state, due to self-annihilation, only *ortho*-Ps atoms were detected by the MCP which was placed at a distance, *L*, of 0.88 m from the target. Although the *m*=0 states of *ortho*-Ps atoms are perturbed and its lifetime becomes shorter by Zeeman mixing with *para*-Ps atoms in a magnetic field, this effect is negligibly small, even in the Ps (*n*=2) state at the present field strength^35^.

### Observation

**Figure 2:**
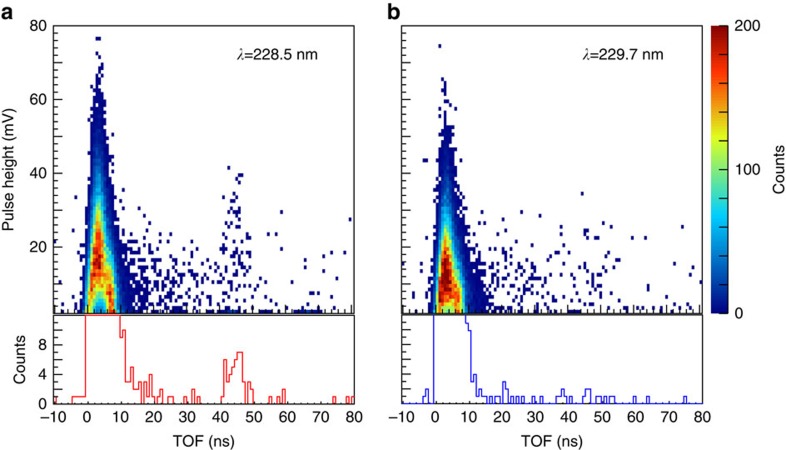
2D time-of-flight spectra of the MCP signals. The wavelengths of the laser beams were 228.5 nm (**a**) and 229.7 nm (**b**). The bottom sections are the vertical projections of the spectra with pulse height over 18 mV. When *λ*=228.5 nm, delayed signals from the detection of Ps atoms formed by photodetachment are observed at *t*=44 ns, while these signals are not observed for *λ*=229.7 nm.

Figure 2 shows the 2D time-of-flight (TOF) spectra of the MCP signals at two different laser wavelengths for *V*=3,400 V, accumulated over 2 × 10^3^ s. The prompt peaks seen at time *t*=0–10 ns are attributed mainly to the detection of stray light. Annihilation γ-rays of the positrons in the target and self-annihilation of *para*-Ps also contribute to these peaks. No significant signal is observed at the laser wavelength 229.7 nm, a delayed peak is seen at *t*=44 ns when the wavelength is tuned to 228.5 nm. The TOF is consistent with that of Ps atoms formed by photodetachment, given by 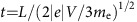, where *e* and *m*_e_ are the charge and the rest mass of the electron, respectively.

**Figure 3:**
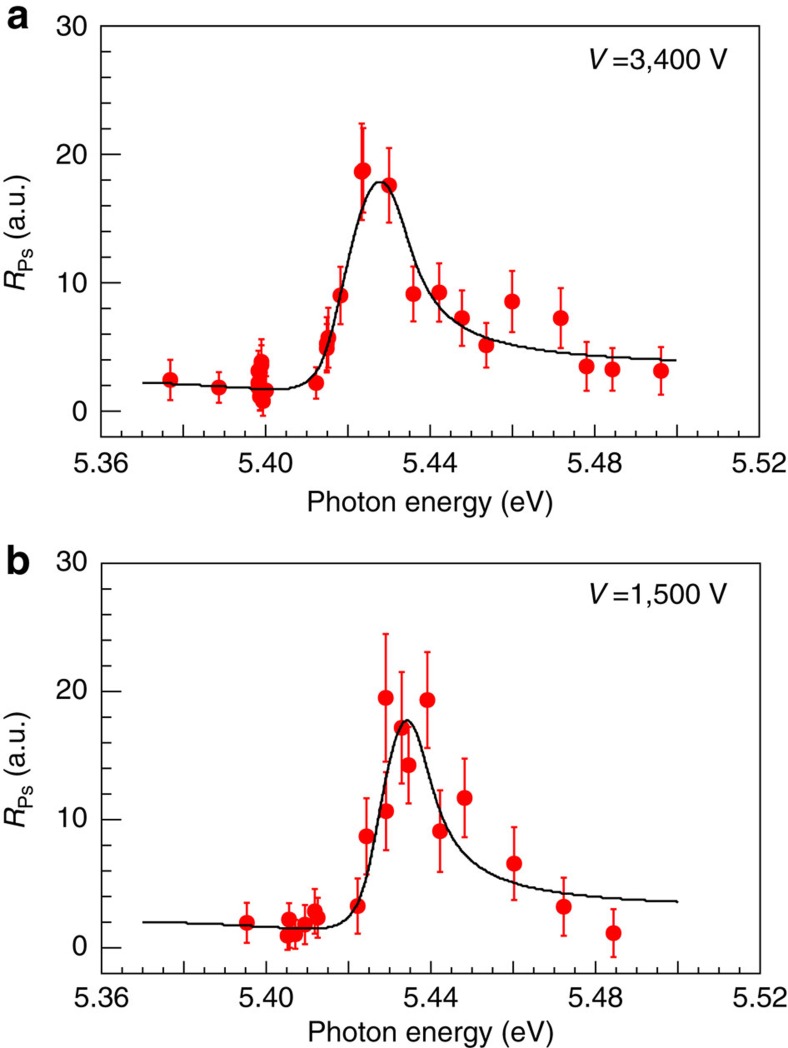
Resonance profiles of Ps^−^ ions in the vicinity of the *n*=2 threshold. *R*_Ps_ plotted against photon energy for acceleration voltages of 3,400 V (**a**) and 1,500 V (**b**). The best fit results using a Fano profile convoluted with a Gaussian profile which represents the angular distribution of Ps^−^ are indicated by the solid lines, where the fitting parameters, except for the resonance energy, were constrained to be the same for both sets of data (*χ*^2^/*v*=0.66). Error bars show the standard deviation of the mean *R*_Ps_ values including the error of normalization factors.

The count rate of the Ps atoms, *R*_Ps_, was determined using *R*_Ps_=*R*_PL_−*R*_P_−*R*_L_, where *R*_PL_ and *R*_P_ are the signal rates with and without the laser irradiation, respectively, for the TOF windows of 40–50 ns (*V*=3,400 V) and 62–72 ns (*V*=1,500 V). R_L_ is the background rate due to the laser irradiation. *R*_Ps_ was normalized to the average photon flux and the overlapping volume of the laser beam and the Ps^−^ beam estimated from each spatial and temporal profile to ensure proportionality to the photodetachment cross sections (Supplementary Figs 1 and 2, and Supplementary Note 2). Figure 3 shows *R*_Ps_ measured as a function of the wavelength from 225 nm (5.51 eV) to 231 nm (5.37 eV) for *V*=3,400 V and *V*=1,500 V. Asymmetric peaks with a tail to higher photon energies were clearly observed in both cases.

## Discussion

The photodetachment cross sections, *σ*(*hv*), near resonances with energy *E*_r_ and width *Γ* are often described by the Fano line profile^36^,


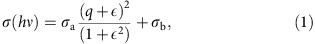


where


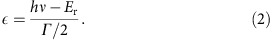


Here, *σ*_a_ and *σ*_b_ are the cross sections of continuum states interacting with and without the resonance state, respectively, and *q* is the shape parameter. It has been reported that the Fano profile describes the shape resonances (^1^P^o^) of H^−^ and D^−^ (refs 37, 38), and was applied to molecular shape resonances^39^. The data obtained were fitted with this profile, as shown in Fig. 3a,b, where the fitting parameters, except for *E*_r_, were kept the same for both cases. *σ*_b_ was assumed to be constant. In the laboratory frame, because of the Ps^−^ motion perpendicular to the average Ps^−^ velocity **v**_***z***_, transverse Doppler-broadening takes place. Accordingly, a Gaussian profile with s.d.=1.3 × 10^−3^*hv*, obtained in a previous measurement^40^, has been convoluted to the fitting profile. The values of *E*_r_ derived by the fitting were 5.4246(12) eV (*V*=3,400 V) and 5.4317(16) eV (*V*=1,500 V), where the errors represent the s.d. of the fitted values. It is clearly seen that each resonance position shifts with *V*, due to the longitudinal Doppler effect expressed as 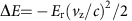. The zero-velocity values of each *E*_r_ extracted from this formula, 5.4367(12) eV (*V*=3,400 V) and 5.4370(16) eV (*V*=1,500 V), are consistent within the s.d. Therefore the resonance energy in the rest frame of the ions was deduced to be 5.437(1) eV from the weighted arithmetic mean of these values. *E*_r_ and the other fitting parameters are listed in Table 1, along with theoretically derived values of the shape resonance by the adiabatic treatment^9^, the complex rotation method^10^ and the hyperspherical close-coupling method^12^. The obtained *E*_r_ and *Γ* values are in good agreement with the theoretical predictions to within meV precision. The shape parameter *q* is also consistent with the theoretical value obtained by fitting the Fano profile to the photodetachment cross sections in the (ref. 12).

In conclusion, we have developed an experimental system for Ps^−^ laser spectroscopy based on an efficient Ps^−^ source. We have observed the ^1^P^o^ shape resonance in the photodetachment of Ps^−^ ions near the *n*=2 threshold. The present experimental resolution is constrained by the Doppler width of about 7 meV due to the Ps^−^ motion. With a combination of the present Ps^−^ production system and the two-photon absorption technique, in which the Ps^−^ ions are irradiated with two counter-propagating laser beams to cancel the Doppler shift, the observation of the narrower Feshbach resonances^8^,^41^,^42^ will be feasible. This precise spectroscopy will be the next challenge for future research.

## Methods

### Laser system

The light source was based on a nano-second dye laser (Sirah, Cobra-Stretch-D; dye solution: Coumarin 460) pumped by the third harmonic of a Q-switched Nd:YAG laser with a repetition of 10 Hz. In order to extend the dye lifetime, DABCO (1, 4-diazabicyclo [2.2.2] octane) was dissolved in the dye solution at 1 g l^−1^ (ref. 43), thereby, almost tripling the lifetime. The outputs were converted to the second harmonics by a type I BBO crystal, resulting in a wavelength range of 225–230 nm with a nominal linewidth of about 0.4 pm (9 μeV). The wavelength was measured using a wavelength metre (HighFinesse, WS-6). The average pulse-width of the output pulses was about 10 ns FWHM, and the average energy was measured to be several 10^−4^ J by an energy metre (Coherent, J-25MUV-193). The spatial and temporal profiles of the laser beam were continuously monitored by a beam profiler (Thorlabs, BC106-UV) and a photodiode (Thorlabs, DET10A/M), respectively. The polarization of the light was set to be parallel to the Ps^−^ velocity vector.

### Data acquisition

The waveforms of the MCP signals were recorded by a digitizer with a 10-bit resolution (National instruments, PXIe-5162). The sampling rate and the band width were 1.25 GS s^−1^ and 1.5 GHz, respectively. The characteristic properties of the laser beam (wavelength, energy, spatial profile and temporal profile) were recorded in synchronization with the digitizer. Data, with and without laser, were recorded with the repetition ratio of positrons (50 Hz) and laser (10 Hz).

### Effect of positronium atoms in *n*=2 excited states

For the measurement of the resonance profile, presented in Fig. 3a,b, above the *n*=2 threshold (5.428 eV), Ps in the *n*=2 state is formed in competition with the *n*=1 state. As for the 2^3^P states, they are de-excited to the 1^3^S state (Lyman-α transition) within a lifetime of 3.2 ns before reaching the MCP detector, while most of the Ps in the metastable 2^3^S state can reach the detector without in-flight loss since the annihilation lifetime of this state is ten times longer than that of the 1^3^S state and de-excitation is forbidden. The detection efficiencies of the 2^3^S state are thus 1.3 times and 1.5 times higher than those of the other states for acceleration voltages of 3,400 and 1,500 V, respectively. To evaluate this contribution, we multiplied these ratios by 2S partial photodetachment cross sections calculated by the HSCC method^12^ and compared them with the total photodetachment cross sections with and without the multiplication. We found a shift of resonance energy of only 0.2 meV when it was taken into account, therefore this effect was disregarded.

## Additional information

**How to cite this article:** Michishio, K. *et al*. Observation of a shape resonance of the positronium negative ion. *Nat. Commun.* 7:11060 doi: 10.1038/ncomms11060 (2016).

## Supplementary Material

Supplementary Supplementary Figures 1-2, Supplementary Notes 1-2 and Supplementary References.

## Figures and Tables

**Table 1:** Comparison of experimental and theoretical results for the ^1^P^o^ shape resonance in the vicinity of the *n*=2 threshold.

	Experiment	Theory		
	Present	Botero *et al*.^9^	Bhatia *et al*.^10^	Igarashi *et al*.^12^
*E*_r_ (eV)	5.437 (1)	5.44	5.438 (1)	5.4375
*Γ* (eV)	0.010 (2)	0.01	0.012 (1)	0.013
*q*	3.9 (8)			3.65*

*E*_r_, resonance energy; *Γ*, resonance width; *q*, shape parameter.

Errors of the experimental values represent s.d. of the fitted values.

The resonance energy in the theory was derived with reference to a ground state energy of −7.1295208, eV (ref. 23).

^*^The shape parameter was obtained by fitting a Fano line profile to the total photodetachment cross sections of the shape resonance in the (ref. 12).
