# Supplementary material for: Observation of a shape resonance of the positronium negative ion
Source: Nat Commun. 2016 Mar 17;7:11060. doi: 10.1038/ncomms11060 (PMC4800431; doi:10.1038/ncomms11060)
Supplement: Supplementary — Figures 1-2, Supplementary Notes 1-2 and Supplementary References. [file ncomms11060-s1.pdf]

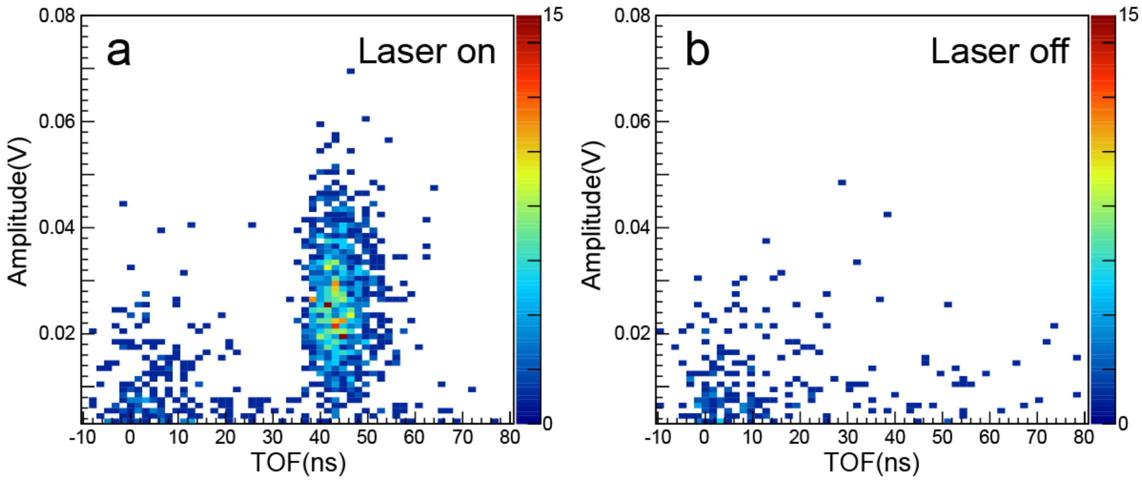

Supplementary Figure 1. 2D time-of-flight spectra from the MCP signals for laser on (a) and off (b), with a Ps^- acceleration voltage of 3400 V.

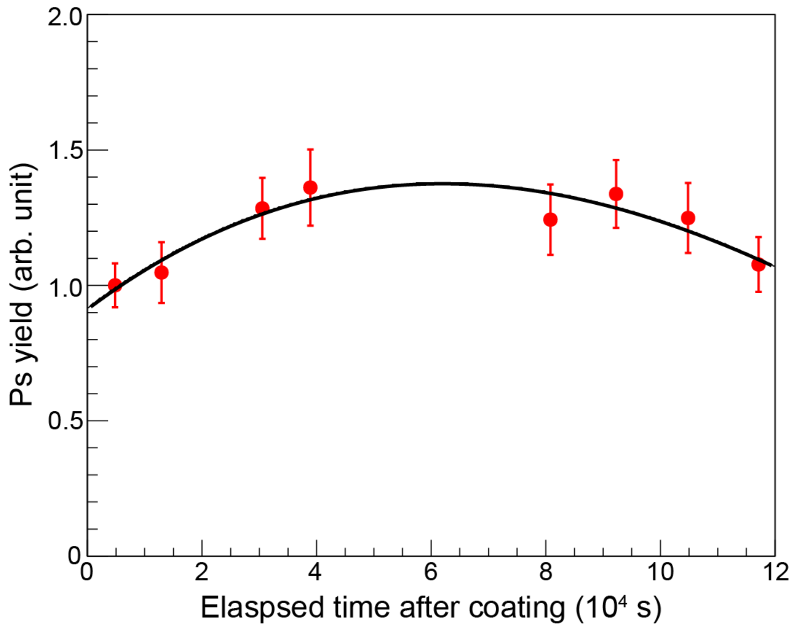

Supplementary Figure 2. Time variation of the Ps yield formed by photodetachment after the Na-coating. Error bars indicate the s.d. of the detected event numbers.

Supplementary Note 1
Sample preparation

In this experiment, a Na-coated tungsten (W) surface was used to produce the positronium negative ions (Ps^-). The Ps^- formation efficiency on W surface is significantly improved by an alkali-metal coating; the effect of Na-coating W on Ps^- emission is discussed in detail elsewhere[1]. The Ps^- production target used was a poly-crystalline tungsten foil of thickness 50 μm (purity 99.95 %) and size of 3×3 mm^2. In order to remove bulk defects (which act as positron traps) and surface contamination such as oxide layers, the target was annealed *in-situ* by passing an electric current through it in vacuum at a base pressure of 1×10^{-8} Pa. The temperature, monitored by a pyrometer, was raised to about 1800 K and held there for 20 min. Although a temperature of 2600 K is required to remove most of the vacancy-type defects[2], the highest temperature used was limited to 1800 K in order to avoid deforming the target. It has been found that efficient formation of Ps^- ions can be obtained using this annealing temperature[3]. After cooling to room temperature, the W surface was coated with Na atoms using commercially made Na dispensers (SAES Getter S.p.A) placed at a distance of about 0.3 m from the target. The thickness of the layer, which was monitored by a crystal oscillator (INFICON, UHV bakeable sensor), was set to be about 0.3 monolayer, where a relatively high Ps^- formation efficiency has been previously reported (~2%)[3].

Supplementary Note 2
Normalization of the experimental data

・Correction for the variation of the Ps^- formation efficiency

The formation efficiency of Ps^- ions varies gradually over time due to residual gas adsorption onto the Na-coated tungsten surface. Therefore, the data shown in Fig. 3 of the main manuscript has been corrected accordingly for the yields of Ps atoms formed by photodetachment using a high-power fundamental wave (1064 nm) from a Q-switched Nd:YAG laser (Spectra Physics, GCR290) before and after each ultraviolet (UV) measurement with the dye laser. The repetition rate of the Nd:YAG laser was 25 Hz, which was half of that of the positrons. The laser beam was shaped by a mirror with

a central aperture, resulting in a circular beam of waist diameter 4 mm and energy 60 mJ. The timings of the laser and the positron beams were monitored using a photomultiplier tube (Hamamatsu Photonics, H6614) coupled with a plastic scintillator. This simultaneously detected the annihilation γ-rays and laser beams entering a pin-hole in the light-tight wrapping.

Supplementary Figure 1 shows typical two-dimensional time-of-flight spectra from the MCP signals accumulated over 1000 s, for a Ps^- acceleration voltage of 3400 V, with and without laser irradiation. Prompt peaks at t =0-10 ns originate from the detection of annihilation γ-rays from the target and short-lived para-Ps atoms. A peak attributed to Ps formed by photodetachment is seen around t =44 ns. The typical count rate of Ps atoms was 1 cps with an amplitude threshold of 6 mV and the TOF windows of 35-55 ns. The time variation of the Ps yield is plotted in Supplementary Figure 2. The yield increased gradually after the Na-coating and then decreased. This behavior is similar to that observed in previous measurements of the yields of the Doppler-shifted annihilation γ-rays from accelerated Ps^- ions[3]. The data was fitted to a low-order polynomial as indicated by the solid line in Supplementary Figure 2. The interpolated values were used to correct the experimental data obtained in the UV measurement.

・Correction for the overlapping volume of the Ps^- and the UV laser beams

Overlapping volumes of the Ps^- and the UV laser beams have been evaluated to correct the data for the variation of the spatial profile of the laser beam. The Ps^- ions travel in the z direction (say), pass through the forward grid at z=0 and intersect with a laser beam in the x direction. Then the overlapping volume, h, is given by a convolution integration of overlap integration between each spatial profile with time, t, as

$$h = \int_{-\infty}^{\infty} dt \int_{-\infty}^{\infty} dx \int_{-\infty}^{\infty} dy \int_{0}^{\infty} dz\, N_{\mathrm{p_{xy}}}(x,y) N_{\mathrm{p_z}}(z - vt) N_{\mathrm{l_{yz}}}(y,z) N_{\mathrm{l_x}}(x - ct) e^{-z/v\tau}, \quad (1)$$

where $N_{\mathrm{p_{xy}}}(x,y) N_{\mathrm{p_z}}(z - vt)$ and $N_{\mathrm{l_x}}(x - ct)\, N_{\mathrm{l_{yz}}}(y,z)$ are the spatial profiles of the Ps^- ions and the laser beam normalized as

$$\int_{-\infty}^{\infty} dx \int_{-\infty}^{\infty} dy \int_{-\infty}^{\infty} dz\, N_{\mathrm{p_{xy}}}(x,y) N_{\mathrm{p_z}}(z) = 1,\ \int_{-\infty}^{\infty} dx \int_{-\infty}^{\infty} dy \int_{-\infty}^{\infty} dz\, N_{\mathrm{l_x}}(x) N_{\mathrm{l_{yz}}}(y,z) = 1. \quad (2)$$

The speeds of the Ps^- ion and light are denoted by v and c, respectively, and τ is the Ps^- lifetime (479 ps). Here, the rate of the photodetachment process induced by laser

irradiation at the wavelength of the shape resonance (expected to be one order of magnitude lower than that of self-annihilation[4]) is ignored. We assumed that the 2D profile of Ps^-, $N_{p_{xy}}(x, y)$, is the same as that of the positron beam because the flight pass length of the Ps^- ions from the target to the laser intersection point is only 4 mm and they are accelerated by a parallel electric field. Therefore, $N_{p_{xy}}(x, y)$ was measured by the positron beam impact onto a MCP coupled with a phosphor screen placed at the target position. The 2D laser profiles, $N_{l_{yz}}(y, z)$, were measured using a beam profiler (Thorlabs, BC106-UV). The 1D profiles of $N_{l_x}(x)$ and $N_{p_z}(z)$ were obtained from the temporal profiles of the laser and the Ps^- (positron) beams, measured using a photodiode (Thorlabs, DET10A/M) and a photomultiplier tube (Hamamatsu Photonics, H6614) coupled with a plastic scintillator, respectively.

Supplementary References

1. Nagashima, Y. Experiments on positronium negative ions. *Phys. Rep.* **545**, 95-123 (2014).
2. Debelle, A., Barthe, M. F. & Sauvage, T. First temperature stage evolution of irradiation-induced defects in tungsten studied by positron annihilation spectroscopy. *J. Nucl. Mater.* **376**, 216-221 (2008).
3. Terabe, H., Michishio, K., Tachibana, T. & Nagashima, Y. Durable emission of positronium negative ions from Na- and K-coated W(100) surfaces. *New J. Phys.* **14**, 015003 (2012).
4. Igarashi, A., Shimamura, I. & Toshima, N. Photodetachment cross sections of the positronium negative ion. *New J. Phys.* **2**, 17 (2000).
